# Study on Retrofitted Masonry Elements under Shear Using Digital Image Correlation

**DOI:** 10.3390/s20072122

**Published:** 2020-04-09

**Authors:** Benjamín Torres, Francisco B. Varona, F. Javier Baeza, David Bru, Salvador Ivorra

**Affiliations:** Department of Civil Engineering, University of Alicante, 03080 Alicante, Spain; benjamin.torres@ua.es (B.T.); borja.varona@ua.es (F.B.V.); david.bru@ua.es (D.B.)

**Keywords:** digital image correlation (DIC), masonry, shear behavior, fiber-reinforced cementitious matrix (FRCM), textile-reinforced mortar (TRM)

## Abstract

Architectural heritage is usually built with masonry structures, which present problems under lateral in-plane loading conditions, such as wind pressure or earthquakes. In order to improve the shear behavior of masonry, the use of a fabric-reinforced cementitious matrix (FRCM) has become an interesting solution because of its synergy of mechanical properties and compatibility with masonry substrates. For a proper structural evaluation, the mechanical behavior of reinforced masonry and the FRCM itself needs to be characterized. Hence, a numerical model to evaluate the FRCM reinforcement requires some mechanical parameters that may be difficult to obtain. In this sense, the shear behavior of masonry can be evaluated by means of diagonal tension tests on small specimens (71 × 71 cm). In this work, a digital image correlation (DIC) monitoring system was used to control displacements and cracking patterns of masonry specimens under shear stress (induced by diagonal tension with FRCM layers) applied to one or two sides. In addition, the mechanical behavior of FRCM coupons under uniaxial tensile tests was also registered with DIC. The displacement measurements obtained by DIC were validated with the measurements registered with LVDT. Unlike LVDT-based techniques, DIC monitoring allowed us to measure deformations in masonry during the full test, detecting crack initiation even before it was visible to the eye.

## 1. Introduction

Much of our architectural heritage has been built in stone or brick masonry. In many cases, there were hardly any structural design calculations behind these constructions, but in those occasions in which the design was based on some numerical justifications, these only included the effect of gravitational loading. Accordingly, the usual structural typologies are especially sensitive to lateral loads, like the shear forces associated with wind pressure or seismic events [1,2,3]. Therefore, most of them exhibit different pathologies as a consequence of the lack of reinforcement to resist the tensile stresses induced by lateral loading conditions [4,5]. Furthermore, special considerations should be made in slender and more vulnerable structures such as chimneys [6,7] and bell towers [8,9]. If structural retrofitting or strengthening are required, modern composite materials present a very attractive solution because the combination of a fiber reinforcements inside a cement or polymer matrix can be tailored to meet the specific requirements of each structure [10,11]. The use of a fabric-reinforced cementitious matrix (FRCM)—also referred as textile-reinforced mortar (TRM)—embodies a more-than-adequate solution for the retrofitting of masonry elements, because of its better compatibility and reinforcement permeability [12,13,14] compared to fiber-reinforced polymers (FRP).

For a proper structural evaluation, the mechanical behavior of masonry needs to be characterized at different scales, i.e., structural modal analysis and the mechanical properties of materials. Hence, a combination of both approaches is required to define a numerical model for a vulnerability study. Dynamic testing can be carried out to determine the modal response (frequencies and modal shapes) of the whole structure [15]. Modal updating techniques are used to adjust the mechanical parameters of the structure and to calibrate the modal response of the numerical model [16]. Traditional contact measures using accelerometers could be an accurate solution, provided that vibrations are generally small and nonlinear effects—like relevant cracking—are not triggered. In this case, contactless techniques based on image analysis offer some advantages when accessibility to inspection points is difficult or even impossible [17,18,19] (e.g., the exterior surface of brick chimneys).

On the other hand, from the material point of view, the shear behavior of masonry may be studied with medium-scale samples subjected to diagonal tension tests [20,21]: A masonry specimen of square shape is loaded in compression along one diagonal, and the shear strain is measured during the full test. The shear strain is obtained through the longitudinal strains along both diagonals, and, in order to measure these deformations, current standards recommend the use of LVDTs [22]. However, this type of instrumentation presents some experimental limitations like the risk of breaking the LVDTs if masonry presents sudden and brittle failure. Furthermore, if the gage lengths of the LVDTs are insufficient, masonry cracking may occur beyond the measurement area, and the experimental observations would not represent the actual behavior of the specimen under shear stress. Besides, the only available information would be an average elongation or shortening of the gauge length, but crack initiation and growth would not be characterized. Advanced numerical modelling to consider nonlinear effects in masonry or FRCM reinforcements includes mechanical parameters such as crack separation or fracture energy [14,23]. The latter require specific data related to cracking phenomena. Hence, digital image correlation (DIC) systems constitute an interesting alternative to monitor strains and displacements based on a non-contact optical technique [24,25,26,27].

DIC has been widely used to experimentally obtain displacements and strains in concrete structures [28,29,30], usually by means of electrical sensors. In this regard, this non-contact technique has multiple advantages for global element monitoring, as it allows for the control of the entire photographed element, beyond the sensor installation area. In addition, the implementation of this technique may be reduced to a single digital imaging camera, which considerably simplifies the experimental test setup. Compared to structural concrete elements, the monitoring of masonry structures presents researchers with additional challenges that make it necessary to look for innovative techniques, e.g., embedded smart materials or thermography among other systems [17,31]. In this regard, the application of DIC in monitoring masonry structures is less widespread [32,33], and its potential application to the characterization of masonry material has been focused on the evaluation of different reinforcements [26,34] rather than the behavior of the unreinforced masonry itself. An additional advantage of DIC is that it makes it possible to register measurements after crack initiation. This feature overcomes some drawbacks of traditional LVDT transducers, which have to be removed at some point when testing brittle materials and whose sudden failure may eventually break the LVDTs. In addition, the combination of DIC and traditional sensors in laboratory testing may improve the understanding of the mechanical behavior of composite reinforcements, as well as the identification of their properties [35].

Furthermore, the interpretation of monitoring data in masonry structures is sometimes difficult because the heterogeneity of the materials can interfere with the installation of sensors. The existence of mortar and brick joints sometimes makes the behavior unpredictable, and failure occurs unexpectedly. Hence, the sensor may not provide useful information because the failure occurred in a different area than the one initially expected. In this matter, DIC offers the possibility of knowing the behavior of the entire photographed element and the full strain field. As has been commented above, the use of DIC in masonry provides very relevant information about the cracking process that conventional sensors do not provide. DIC makes it possible to identify areas of stress concentration, even with low load levels. In the case of unreinforced masonry, the failure follows the mortar joints, whilst in reinforced masonry elements, cracks appear more distributed, generally along the directions of principal tensile stresses. In spite of this, the application of DIC to characterize the behavior of masonry elements is scarce. A few studies have used DIC to study small scale samples with only two or three bricks. For example, the behavior of mortar-less joints in compression was found to depend on the roughness and non-flatness of the brick faces that are in contact [33]. Another work [32] focused on the shear behavior of mortar joints in unreinforced masonry, in which specimens with only three bricks were used to characterize the fracture mechanisms of mortars with different properties.

The current study was aimed at the characterization of full-scale masonry elements using DIC. Usually, DIC monitoring systems require costly equipment and data post-processing [30,36]. Nonetheless, in this work, a low cost system that was used to monitor the shear behavior of masonry walls and FRCM coupons is proposed. The tests were carried out in accordance with the standard for diagonal tension testing using masonry specimens with and without FRCM reinforcement. As a secondary objective, the behavior of FRCM coupons under uniaxial tensile loading was also measured using DIC. The result of the DIC measurements was compared to conventional monitoring systems using LVDTs in order to highlight the advantages and limitations of DIC for practical applications. With this procedure, the response of numerical models of masonry structures could be calibrated, and a similar monitoring system may be used for testing or monitoring real-scale structural masonry elements.

## 2. Materials and Methods

The masonry test specimens were made of solid clay bricks, and the joints were made of lime mortar. The bricks were 230 × 110 × 55 mm in size, with a density of 1550 kg/m^3^ and a compressive strength of 15 MPa (according to the manufacturer’s specifications). Nevertheless, the strength was also measured according to the standard UNE-EN 1015-11:2000/A1:2007 [37], obtaining an average value of 17.2 at 28 days (with a coefficient of variation of 15%). The lime mortar used to make the masonry joints was of the Morcem Cal Muro type, supplied by Puma Group. This mortar contained mainly natural hydraulic lime with natural pozzolan, aggregates, and additives. According to the supplier, its compressive strength was equal to or greater than 7.5 MPa—which was also experimentally obtained at 28 days, resulting in 9.4 MPa with a coefficient of variation of 12%.

In order to improve the in-plane shear strength of the masonry elements, several test specimens were retrofitted with a layer of TRM applied on one side or on both sides. The mortar was of the Planitop HDM Restauro type, reinforced with 12 mm discrete glass fibers, supplied by Mapei. It was a pre-blended two-component mortar with high ductility, based on hydraulic lime and eco-pozzolan, with a compressive strength of at least 15 MPa after 28 days. The textile reinforcement of the mortar consisted of a G220 glass fiber-reinforced polymer mesh supplied by Mapei. The pitch of the mesh was 25 × 25 mm, and its approximate mass was 225 g/m^2^. The supplier specified the following mechanical properties: a longitudinal modulus of elasticity of 72 GPa, an ultimate strength of 1276 MPa, and an ultimate elongation of 1.8%. 

**Figure 1:**
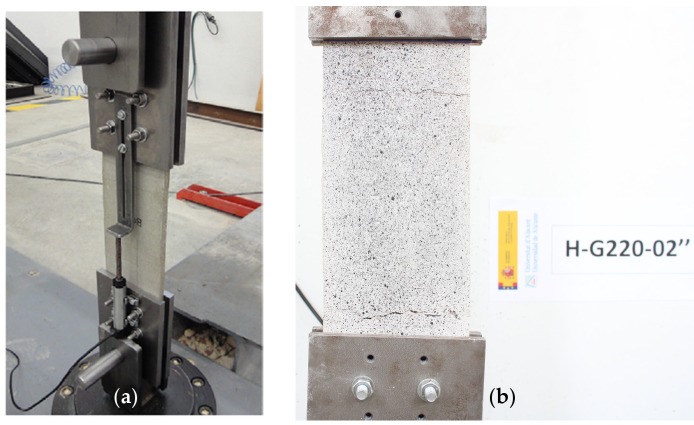
Direct uniaxial test of a fabric-reinforced cementitious matrix (FRCM) coupon: (**a**) experimental setup; (**b**) crack pattern.

The mechanical performance of the TRM was tested in accordance with Annex A of standard AC434 ‘Tensile Testing of Fiber-Reinforced Cementitious Matrix (FRCM) Composite Specimens’ [38]. Three coupons were fabricated with dimensions 400 × 100 × 9 mm (length × width × thickness) and tested at an age of 60 days. Each FRCM coupon was bolted to steel clamps installed in an electro-mechanical press with a loading cell of 50 kN. The test velocity was set at 0.2 mm/min, and the elongation was measured with one LVDT, as well as DIC. The experimental configuration is shown in Figure 1.

**Figure 2:**
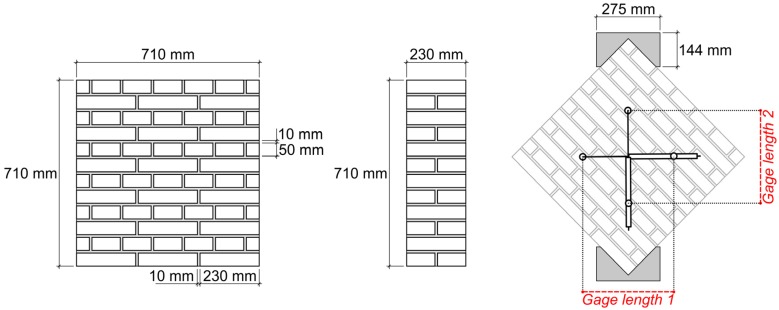
Masonry specimens for the diagonal tension test: dimensions and experimental setup.

**Figure 3:**
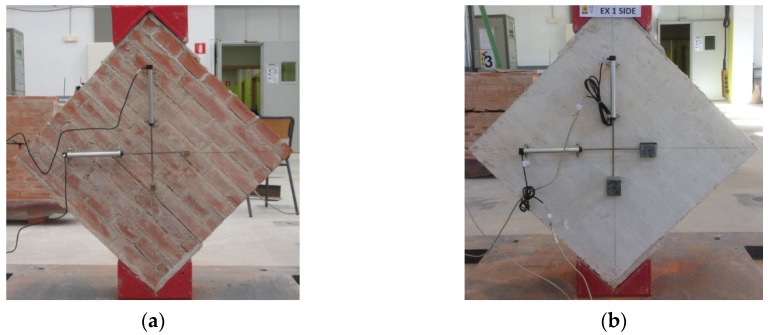
Installation of the LVDT devices to measure diagonal deformation: (**a**) installation on an unreinforced masonry specimen; (**b**) installation on an FRCM-reinforced masonry specimen.

On the other hand, the shear behavior of the masonry specimens was measured through diagonal tension tests in accordance with standard ASTM E519/E519M-15 ‘Standard Test Method for Diagonal Tension (Shear) in Masonry Assemblages’ [22]. The size of each masonry sample was 710 × 710 × 230 mm (see Figure 2). The test was displacement-controlled with a velocity of 0.54 mm/min. Two LVDTs were installed on one face of each specimen, one along each diagonal direction (Figure 3). Based on the specimen’s deformations (obtained along the 43 cm gage lengths in Figure 2) the shear stress vs. shear strain relationship was determined. Further details are given in the ‘Results’ section.

Six masonry specimens were built for the shear behavior characterization: Two of them were unreinforced, another two were reinforced with an FRCM on one side only, and the other two were reinforced on both sides. The FRCM reinforcement was installed when the specimens had an age of 28 days, and the diagonal tension tests were carried out at an age of 90 days. Prior to the test, one of the unreinforced masonry specimens was subjected to the shaker simulation of vibration-induced damage. For this purpose, an LDS V406 permanent magnet shaker was used, with a mass of 5 kg excited with white noise during 500 h. The mass oscillated vertically with respect to the masonry specimen.

**Figure 4:**
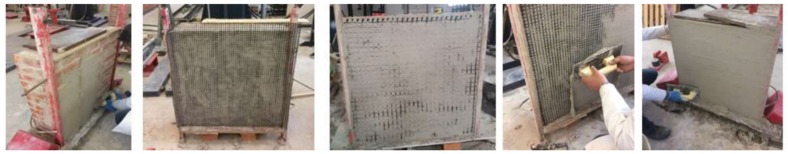
Illustration of the retrofitting of masonry specimens with textile-reinforced mortar (TRM) in the laboratory.

The construction procedure for the FRCM-strengthening is illustrated in Figure 4. A mortar layer was placed upon the surface of the masonry specimen to regularize the surface. The thickness of the mortar layer was approximately 5 mm. Then, when the mortar was still in its fresh condition, the reinforcing glass fiber-reinforced polymer mesh was applied. Next, a second layer of mortar was spread up to a maximum total thickness of 1 cm. Finally, the surface was evened out with a trowel.

### DIC Measurements

DIC systems are based on greyscale value digital images that have been captured using a digital camera. Using a stereoscopic sensor setup, each object point is projected onto a specific pixel in the image plane of the sensor [39]. A reference length is necessary to define the absolute size and scale of the object. The position of each point in the image can be identified by applying a correlation algorithm using a stochastic intensity pattern (speckle) onto the specimen’s surface. The displacement of each point of the speckle allows the program to compute the full field of displacements and strains. Thus, the elongation measures are not limited to the gage length of the LVDT or the exact position of a strain gauge. Accordingly, the full displacement distribution or local strains can be obtained. Another advantage of this technique is the characterization of the crack patterns. The main limitation is the actual resolution of the DIC, which depends on the pixel density of the camera, the size of the area of interest, and the quality of the speckle. Resolution and accuracy are associated with the resolution of the optical sensor installed in the camera. As a first approximation, the method provides measurements with the resolution of the size of one pixel, but sub-pixel interpolation algorithms could lead to a resolution of 1/50th or even 1/100th of the pixel [35]. Finally, the displacement field directly obtained by this procedure is discrete, and it is then interpolated with predefined shape functions (e.g., bi-cubic spline [40]) to obtain a continuous field.

For the current DIC measures, a Canon Eos2000D 24 MP camera and the GOM Correlate software were used. Most applications deal with in-plane (2D) displacements, but advances in computers’ computation performance have recently made it possible to also register the tri-dimensional displacement field of a specimen’s surface [36]. Nonetheless, in these tests, only one camera was used because the out-of-plane displacements were considered negligible compared to the in-plane components, based on prior research [41].

**Figure 5:**
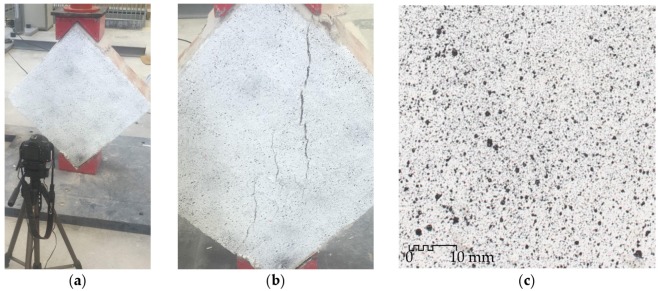
Installation of the digital image correlation (DIC) system to monitor shear behavior of masonry elements during diagonal tension tests: (**a**) experimental DIC setup; black speckle over the surface of (**b**) a masonry specimen reinforced with an FRCM and (**c**) an FRCM coupon in uniaxial tensile tests.

In this measurement technique, a crucial step is the creation of the stochastic pattern [39]. In order to optimize the DIC analysis, images with great contrast are required. This can be achieved by painting the masonry specimens white and subsequently applying a black speckled pattern (Figure 5). The size of the spots in the speckle has a notorious impact on the accuracy of the results, so a suitable balance needs to be defined based on specific setup conditions. An example of the speckle used in these tests can be found in Figure 5c. The spray application made a random particle dispersion, with a maximum particle size of approximately 1.5 mm.

Additionally, pairs of artificial markers (marks made with a red pen spaced a certain distance) were painted on the specimen’s surface, and their displacement was measured by DIC. The high-resolution photographs were taken every 5 s with the camera positioned at 1 m from the specimen. It was not necessary to use any external lighting, since the laboratory lighting during the tests was sufficient to guarantee the correct development of the tests. Pictures had a size of 6000 × 4000 pixels, corresponding to a pixel size of 0.01 mm. Sub-pixel interpolation led to a displacement resolution in the order of 1–2 µm. Digital images were processed with GOM Correlate software program. The stress–strain response curves were derived separately based on the data provided by the two displacement/strain measurement methods—DIC and LVTDs—and subsequently compared to each other to validate the results.

## 3. Results

In the presentation of results and the following discussion, the following notation is used:

- E_f_^*^ is the elastic modulus of FRCM coupons subjected to direct tensile tests, corresponding to the uncracked state and calculated with respect to the gross cross-sectional area.
- *E_f_* is the elastic modulus of FRCM coupons subjected to direct tensile tests, corresponding to the post-crack condition and calculated with respect to the area of the glass fiber mesh.
- *τ* is the shear stress of masonry specimens in the diagonal tension tests.
- *γ* is the shear strain of masonry specimens in the diagonal tension tests.
- *P* is the vertical load applied in the diagonal tension tests.
- *A_n_* is the net area of the masonry specimen subjected to diagonal tension tests.
- ∆*y* is the average shortening in the direction parallel to the load in the diagonal tension tests.
- ∆*x* is the average extension in the direction perpendicular to the load in the diagonal tension tests.
- *g* is the gage length in the diagonal tension tests.
- *G* is the shear modulus of elasticity, obtained in the diagonal tension tests.
- $\tau_{max}$ is the peak shear stress in the diagonal tension tests.
- ε is the longitudinal strain of FRCM coupons in the direct tensile tests.
- $\varepsilon_{h}$ is the horizontal strain of masonry specimens measured by DIC in the diagonal tension tests.
- ∆P is the strength increase of reinforced masonry in the diagonal tension tests.
- ${\bar{P}}_{max}^{R}$ is the average peak load of reinforced masonry in the diagonal tension tests.
- ${\bar{P}}_{max}^{U}$ is the average peak load of unreinforced masonry in the diagonal tension tests.
- *d_max_* is the displacement at maximum load level in the diagonal tension tests.
- *d_u_* is the ultimate displacement considered as the post-peak displacement with a 20% load loss with respect to the maximum load.
- *μ* is the ductility factor of masonry-reinforced specimens in the diagonal tension tests.
- ∆*u_u_* and ∆*v_u_* are the horizontal elongation and vertical shortening at ultimate conditions, respectively.
- ∆*u_max_* and ∆*v_max_* are the horizontal elongation and vertical shortening at the maximum load value.

### 3.1. Tensile Behaviour of FRCM Coupons

**Figure 6:**
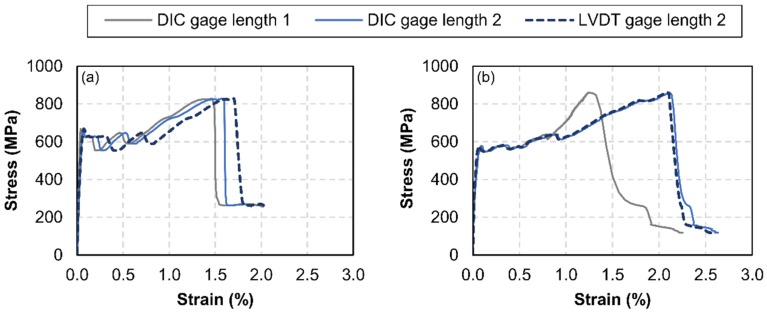
Tensile behavior of the FRCM: stress–strain curves of (**a**) specimen 1 and (**b**) specimen 2. Strains were measured with LVDT and a DIC system.

The experimental results obtained in the uniaxial tensile tests of the FRCM coupons are shown in Figure 6. The horizontal axis corresponds to the longitudinal elongation, and the tensile stress is shown in the vertical axis. The stress was obtained as the axial load divided by the fiber cross-sectional area. Two gage lengths were considered. Gage length 1 was the free distance between clamps, which was approximately 20 cm. This gage length could only be measured through DIC. On the other hand, gage length 2 was used to convert the displacement measured with the LVDT into longitudinal strain, and its length was approximately 30 cm (i.e., the distance between the centroids of the top and bottom bolted coupon-to-clamp connections). Therefore, the increase of gage length 2 could be measured through both DIC and LVDT. Despite three FRCM specimens having been prepared, the test only yielded adequate results in two of them, which are the ones included in Figure 6. The problem with the third specimen was caused by an excessive out-of-plane curvature induced by bending during curing. The loading conditions for this specimen were not those of uniaxial tension but rather those of the flexural-tensile state. This also made it impossible to obtain accurate results with either LVDT of with DIC (because only one camera was available).

The notorious discrepancy that is apparent in Figure 6b between the elongation measurements based on gage length 1 and gage length 2 happened because one crack appeared and grew across the edge of a pair of clamping plates, thus lying beyond gage length 1. Nevertheless, the results obtained with the DIC system showed an excellent agreement with those obtained with LVDT, especially in the case of gage length 2 in Figure 6b; the relative error between the DIC-based and LVDT-based elongation of gage length 2 in Figure 6a was approximately 10%.

These stress–strain relationships made it possible to calculate the longitudinal moduli of elasticity, which are shown in Table 1. The modulus E_f_^*^ corresponds to the uncracked state, and its value was calculated with respect to the gross cross-sectional area of the FRCM coupon (100 × 9 = 900 mm^2^), in accordance with the standard [38]. On the other hand, the modulus *E_f_* corrresponds to the cracked state and was calculated with respect to the cross-sectional area of the glass fiber mesh (3.53 mm^2^), hence its greater value. The relative difference between the DIC and LVDT measurement systems was 6.7% in the case of E_f_^*^ and 1.7%–6.2% in the case of *E_f_*. This error was less than the one associated with the experimental dispersion observed between both specimens, which was close to 11%.

### 3.2. Shear Behaviour of Masonry with Different FRCM Reinforcements

**Figure 7:**
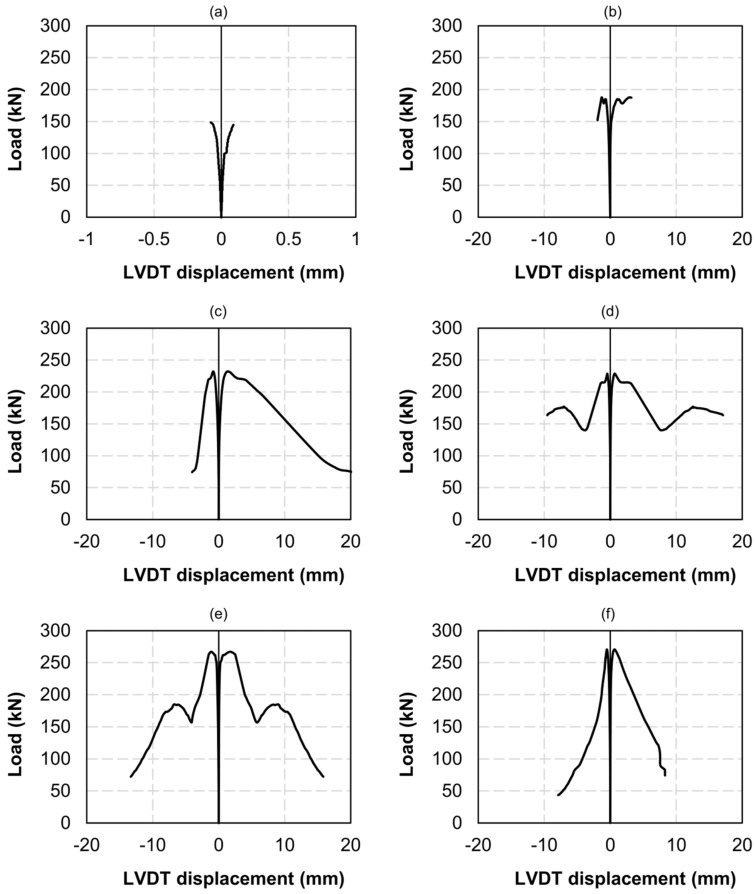
Load–displacement curves for all specimens based on LVDT measurements: unreinforced masonry specimens 1 (**a**) and 2 (**b**); single-reinforced masonry specimens 1 (**c**) and 2 (**d**); double-reinforced masonry specimens 1 (**e**) and 2 (**f**).

Load–displacement curves for all specimens are shown in Figure 7. The horizontal axis corresponds to the displacements measured with LVDT. The LVDTs installed horizontally produced positive displacement, which corresponded to tensile strain. Conversely, the LVDTs installed vertically yielded negative values, which corresponded to compressive strain. The load–displacement curves in Figure 7 reveal that the FRCM-reinforcements notably increased the shear capacity of the masonry specimens, not only in terms of the load applied but also in terms of the ductility. The increase in load capacity was slightly higher in the case of the double-reinforced specimens. It is important to note that the specimens had been reinforced without having been previously subjected to direct loading (although shaker-induced vibrations had damaged the specimen in Figure 7a). Single-reinforced specimens increased their maximum load by 34% with respect to the unreinforced ones, whilst double-reinforced specimens increased it by 52%.

The load and displacements lectures represented in Figure 7 can be used to calculate the shear stress (*τ*) vs. shear strain (*γ*) relationship. In accordance with ASTM E519/E519M-25 [22], the shear stress is calculated through Equation (1):

(1) $$\tau =\frac{0.707\cdot P}{A_{n}}$$

where *P* is the vertical load applied in the diagonal tension test and *A_n_* is the net area of the masonry specimen, which is calculated through Equation (2):

(2) $$A_{n}=\left(\frac{w+h}{2}\right)\cdot t\cdot n$$

where *w*, *h*, and *t* are, respectively, the width, height, and thickness of the specimen (in mm), and *n* is the fraction of cross-sectional area that is solid, i.e., excluding the mortar joints.

On the other hand, the shear strain is calculated through Equation (3):

(3) $$\gamma =\frac{\Delta x+\Delta y}{g}$$

where ∆*y* is the average shortening in the direction parallel to the load, ∆*x* is the average extension in the direction perpendicular to the load, and *g* is the gage length (which was 430 mm in this study; see Figure 2).

**Figure 8:**
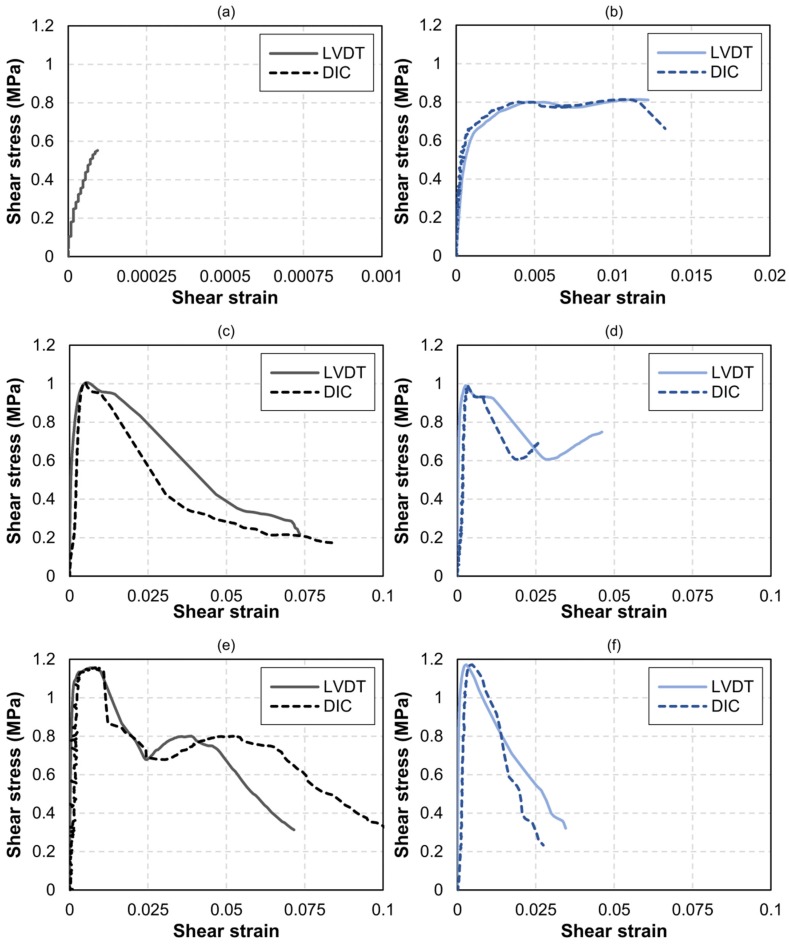
Shear stress–strain curves: unreinforced masonry specimens UM-1 (**a**) and UM-2 (**b**); single-reinforced masonry specimens SM-1 (**c**) and SM-2 (**d**); double-reinforced masonry specimens DM-1 (**e**) and DM-2 (**f**).

The *τ*–*γ* curves are shown in Figure 8. The shear strain *γ* was obtained through Equation (3), and the values of ∆*x* and ∆*y* are those that were registered with both the LVDT devices and the DIC methodology. In the case of the two unreinforced masonry specimens (Figure 8a,b) a large dispersion was observed in terms of both sustained ultimate load and ductility. The specimen shown in Figure 8a (UM-1) had been previously exposed to shaker-induced vibrations for 500 h, which arguably damaged the lime mortar joints and caused the observed brittle behavior. The ultimate shear strain was very small (94 με), and it was not possible to obtain DIC measurements. Besides, this specimen was the first one to be tested, and the high-resolution photographs were focused to capture the whole masonry specimen (including bearings); therefore, the pixel size was found to be too small for the DIC algorithm to accurately detect the strain field. In subsequent tests (Figure 8b,f) the camera focused on a smaller central area of the masonry surface, with a window size similar to the gage length of the LVDTs. 

As can be seen in Figure 8a (specimen UM-1) the *τ*–*γ* relationship was almost linear until failure. Conversely, in the case of the unreinforced specimen in Figure 8b (UM-2), an inelastic behavior developed for shear stresses over 0.7 MPa, resulting in an ultimate shear strain over 1.2%. This inelastic behavior could be attributed to the friction between undamaged joint and bricks. In the case of the single-reinforced specimens (Figure 8c,d) and double-reinforced specimens (Figure 8e,f), notoriously smaller dispersions were observed. Both single-reinforced samples SM-1 and SM-2 reached a shear stress of around 1 MPa, which represented an increase of 25% with respect to UM-2 in Figure 8b. On the other hand, both double-reinforced samples DM-1 and DM-2 reached a shear stress of almost 1.2 MPa, which represented an increase of around 47% with respect to UM-2. The exact results are given in Table 2. 

The agreement between LVDT and DIC measurements was found to be reasonably good up to shear strains *γ* of around 1%. The discrepancy between the DIC-measured shear strains and the LVDT-measured shear strains was significantly larger for *γ* values over 1% in the case of the single-reinforced specimens than in the case of the double-reinforced ones. This was because the lack of symmetry in the reinforcement layout caused a notorious bending of the samples. The LVDTs were installed on the unreinforced surface, which naturally experienced larger displacements with respect to the FRCM-reinforced surface, whose strain field was being captured via DIC. In the case of the double-reinforced specimens, the residual post-crack behavior was accurately characterized with both systems up to shear strain values of around 2%. 

Finally, the shear stress–strain curves that were obtained provided data for calculating the shear modulus of the elasticity *G* of the unreinforced/reinforced masonry through Equation (4):

(4) $$G=\frac{\tau }{\gamma }$$

The *G* modulus is the slope of the initial elastic branch, and its values are given in Table 2. The value *G_LVDT_* corresponds to the shear modulus calculated through LVDT-measured displacements, whilst *G_DIC_* corresponds to the shear modulus calculated via DIC. The discrepancy between them may be attributed to the aforementioned fact that the displacement lectures were not made on the same side.

## 4. Discussion

**Figure 9:**
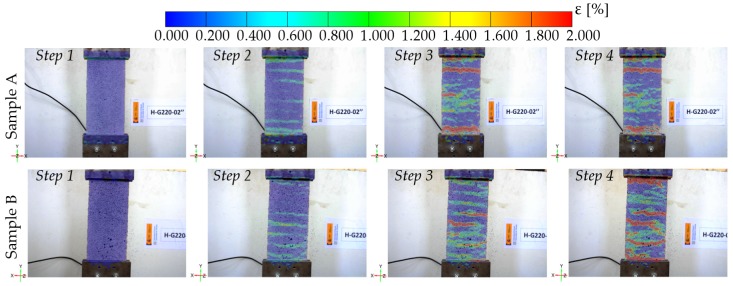
Longitudinal strain distribution at different load levels and crack detection by DIC analysis: step 1 test beginning, step 2 crack initiation, step 3 crack growth, and step 4 excessive crack opening.

**Figure 10:**
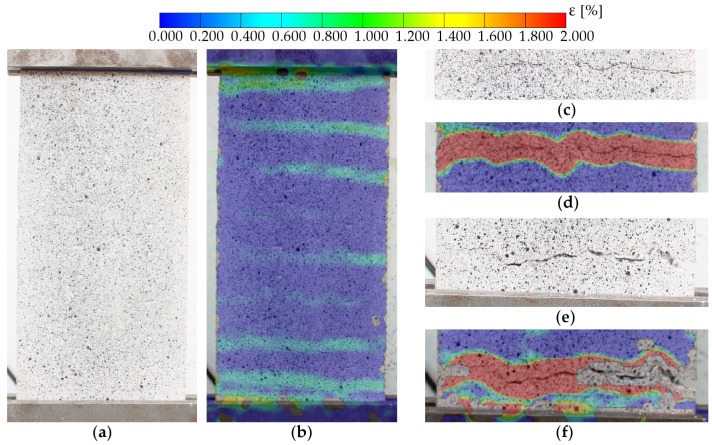
Spatial distribution of transverse cracks in direct tensile tests of sample A in Figure 9. Step 2 corresponding to (**a**) crack initiation and (**b**) crack identification via DIC analysis. Details of clamps in step 4: (**c**) visible crack near the upper clamp and (**d**) crack measurement via DIC; (**e**) non-symmetrical crack growth and (**f**) excessive opening detected by DIC.

Regarding the FRCM uniaxial tests, the measurements with LVDT (Figure 7) served to detect the moment cracking occurs, but their reading could only be used to obtain an average strain. Thus, there was no difference between strains in uncracked zones and crack opening displacements. However, the use of DIC produced a strain distribution through which the crack evolution could be registered. This is illustrated in Figure 9 and Figure 10, in which relevant parameters for the numerical modelling of these materials [1,14] can be obtained, such as the distance between cracks or even the fracture energy. Besides, DIC may have solved some experimental drawbacks that could have occurred in deformation measures with LVDT, like load eccentricity that results in different strains along a cross section of the specimen or an anchorage problem of the fibers or the coupon (which may have led to a slip in the clamps).

**Figure 11:**
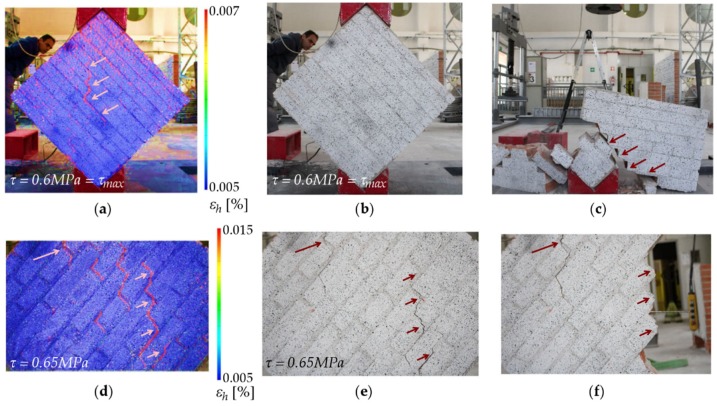
Failure of unreinforced masonry: Specimen (1) (**a**) horizontal strain (%) by DIC at maximum load, (**b**) last image prior to collapse, and (**c**) first image after collapse. Specimen (2) (**d**) horizontal strain (%) by DIC at 0.65 MPa shear stress, (**e**) image prior to collapse, and (**f**) first image after collapse.

In the diagonal tension test of masonry specimens, the DIC monitoring system allowed us to identify the strain distribution at the moment of failure. For example, in one unreinforced sample (Figure 11a–c), the collapse occurred in a very sudden and a brittle way. A main vertical crack (Figure 11a) developed following the mortar joints and failure resulted in a relative slide between the interfaces in the joints of both halves. However, hardly any cracks could be visually detected prior to collapse on either side, despite their registration through DIC. Another example of the velocity of this phenomenon may be observed by comparison of images in Figure 11b,c, which were taken only 5 s apart. On the other hand, the second unreinforced specimen showed a slightly different behavior. The crack propagation along the mortar joints could be observed through DIC (Figure 11a–c). The DIC analysis in Figure 11d was made at a load level corresponding to a shear stress of 0.65 MPa, in which cracks were not yet perceptible to visual inspection. This time, the collapse took longer to take place: 150 s passed between the moment when cracks were already visible (Figure 11e) and the ultimate collapse (Figure 11f).

**Figure 12:**
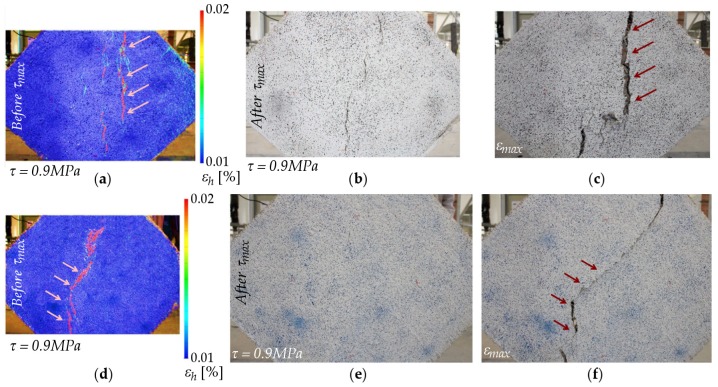
Failure of masonry reinforced in one side: Specimen (1) (**a**) horizontal strain (%) by DIC at 0.9 MPa shear stress before peak load, (**b**) damage at 0.9 MPa shear stress after peak load, and (**c**) damage at maximum test displacement. Specimen (2) (**d**) horizontal strain (%) by DIC at 0.9 MPa shear stress before peak load, (**e**) damage at 0.9 MPa shear stress after peak load, and (**f**) damage at maximum test displacement.

**Figure 13:**
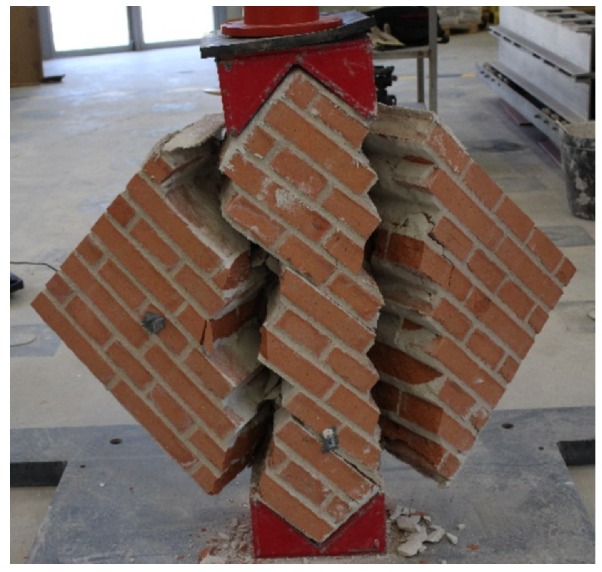
Tilting towards the reinforced in single-reinforced (2) specimen.

In the case of masonry specimens reinforced on one side only, the presence of the FRCM drastically changed the failure mode with respect to unreinforced specimens. DIC images for both tests are included in Figure 12. In both specimens, vertical cracks started to appear between the upper and bottom areas close to the loading shoes, clearly visible on both sides of the specimen. As the loading increased, cracks kept growing along the compressed diagonal, leaving the outer corners unaffected. In addition, the second specimen (Figure 12d–f) started to tilt towards the reinforced side, as already noted in other experimental campaigns [42,43]. This out-of-plane effect (Figure 13), which was only observed in single-reinforced samples, did not result in a ductility reduction. Furthermore, the glass fiber of the FRCM was capable of preventing the total collapse of the masonry, which was observed in the unreinforced specimens.

**Figure 14:**
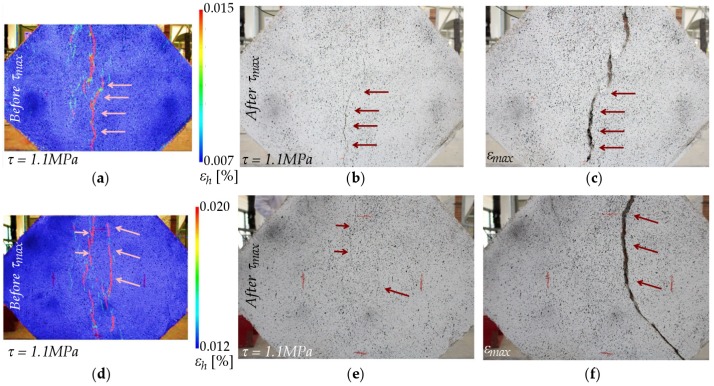
Failure of masonry reinforced in both sides: Specimen (1) (**a**) horizontal strain (%) by DIC at 1.1 MPa shear stress before peak load, (**b**) damage at 1.1 MPa shear stress after peak load, and (**c**) damage at maximum test displacement. Specimen (2) (**d**) horizontal strain (%) by DIC at 1.1 MPa shear stress before peak load, (**e**) damage at 1.1 MPa shear stress after peak load, and (**f**) damage at maximum test displacement.

The failure mode of specimens reinforced on both sides (Figure 14) was characterized by vertical cracks that initially appeared at the center of the specimen. The cracking pattern grew within the upper and bottom areas close to the loading shoes, and a diagonal tension failure occurred in the specimens. The tests did not end with a sudden collapse, i.e., a brittle separation of the two blocks of the specimen, thanks to the use of glass FRCM retrofitting. Both specimens exhibited very similar behavior and achieved a better performance in terms of maximum load and displacement. This enhanced strength and ductility could also be observed through the time that elapsed between the instant when cracks were visible (Figure 14b,e) and the moment when displacement reached its maximum value, which took longer than 10 min.

Finally, besides the aforementioned positive effect of the FRCM reinforcement in the failure of masonry under shear stress, some additional parameters were calculated to quantify the benefits in terms of strength and ductility. The increase of maximum load during the diagonal compression tests was assessed through Equation (5):

(5) $$\Delta P=\left(\frac{{\bar{P}}_{max}^{R}-{\bar{P}}_{max}^{U}}{{\bar{P}}_{max}^{U}}\right)\cdot 100$$

where ${\bar{P}}_{max}^{R}$ and ${\bar{P}}_{max}^{U}$ are the average peak loads for reinforced or unreinforced masonry, respectively. In order to evaluate the ductility, maximum displacement *d_max_* was considered as the displacement at maximum load level, whereas the ultimate displacement *d_u_* was considered as the post-peak displacement corresponding to a 20% load loss with respect to the maximum load [2,44,45]. Therefore, ductility factor *μ* was obtained as the ratio of the ultimate displacement to the maximum displacement, as given by Equation (6):

(6) $$\mu =\min\left\{\frac{\Delta u_{u}}{\Delta u_{max}}\;,\;\frac{\Delta v_{u}}{\Delta v_{max}}\right\}$$

where ∆*u_u_* and ∆*v_u_* are the horizontal elongation and vertical shortening at ultimate conditions, respectively, and ∆*u_max_* and ∆*v_max_* are the same magnitudes measured at the maximum load value. A specimen that experiences inelastic deformations without substantial load-carrying capacity reduction would exhibit a high value of parameter *μ* [42]. 

Table 3 summarizes all the results of Equations (5) and (6) to evaluate the FRCM efficiency as reinforcement solution for the shear behavior of masonry. The application of the FRCM only on one side of the specimen led to a substantial increment (34%) of the load capacity $P_{max}$. This increment was even more important for the symmetrically-reinforced solution, with the FRCM on both sides, in which a 54% strength increase was obtained. In terms of ductility, the unreinforced masonry presented an average ductility factor of 1.2. In this case, FRCM reinforcements increased the ductility factor up to 2.6. However, the highest values were obtained for specimens with only one side reinforced. Despite the fact that experimental tests on masonry usually show a great dispersion of results, three of the four reinforced walls presented fairly identical ductility factors (between 2.4 and 2.6). Therefore, based on the experimental results of this work, a similar ductility enhancement was achieved even with FRCM retrofitting executed on one side only.

## 5. Conclusions

Different masonry specimens and FRCM coupons were prepared and tested to evaluate the effect of FRCM reinforcement on the shear behavior of masonry according to the diagonal tension test in the ASTM standard. The main purpose of this work was aimed at the implementation of a low-cost digital image correlation system for the monitoring of the deformations and strains during the tests. Based on the results and discussion included above, the following conclusions may be drawn.

The FRCM reinforcements were capable of increasing strength and ductility, effectively controlling the failure of masonry in the diagonal tension tests in which a sudden, total collapse was avoided when an FRCM was used.

The displacement measurements obtained by DIC were validated with the values registered with LVDT. Therefore, the shear modulus calculated with both devices was similar. In addition, DIC monitoring allowed us to measure deformations during the full testing of brittle materials, such as unreinforced masonry. DIC monitoring made it possible to detect crack initiation before it was visible to the eye. 

The DIC monitoring system used for this experimental study consisted of a combination of a regular digital camera (without the aid of any special lens or prism), a low sampling frequency (0.2 Hz), and post-processing with free software. Its efficiency on solving the proposed problem was demonstrated, registering information with a high degree of accuracy in full-scale masonry samples. Should this monitoring system be extrapolated to full-scale testing of structural elements with satisfactory results, it could mean a change of paradigm in the current monitoring standards and recommendations for masonry structures and the architectural heritage.

## Figures and Tables

**Table 1:** Longitudinal elastic moduli of FRCM samples obtained with LVDT or DIC.

	Modulus E_f_^*^ [MPa]		Modulus *E_f_* [MPa]	
	LVDT-Based Value	DIC-Based Value	LVDT-Based Value	DIC-Based Value
Specimen 1	6892	7387	31,088	29,274
Specimen 2	7350	6887	26,528	26,090

**Table 2:** Mechanical properties of the masonry specimens subjected to diagonal tension.

	*τ_max_* [MPa]	*G_LVDT_* [MPa]	*G_DIC_* [MPa]
Specimen UM-1	0.64	2049	–
Specimen UM-2	0.81	798	1068
Specimen SM-1	1.00	792	601
Specimen SM-2	0.93	923	693
Specimen DM-1	1.15	1070	1101
Specimen DM-2	1.17	1486	995

**Table 3:** Effect of an FRCM in the shear behavior of masonry: ductility and strength increase.

	μ	$\bar{\mu }$	∆P [%]
Specimen UM-1	1.0	1.2 (26%)	-
Specimen UM-2	1.5		
Specimen SM-1	2.5	2.5 (2%)	34.1
Specimen SM-2	2.6		
Specimen DM-1	1.8	2.1 (20%)	52.0
Specimen DM-2	2.4		
